# Combined Applications of Artificial Intelligence and Simulation for Healthcare Process Optimization: A Systematic Review

**DOI:** 10.3390/healthcare13222933

**Published:** 2025-11-16

**Authors:** Jaime Álvarez-Vázquez, Manuel Casal-Guisande, Alberto Fernández-García, Mar Mosteiro-Añón, María Torres-Durán, Alberto Fernández-Villar

**Affiliations:** 1 Fundación Pública Galega de Investigación Biomédica Galicia Sur, Hospital Álvaro Cunqueiro, 36312 Vigo, Spain; jaime.alvarez@iisgaliciasur.es; 2 NeumoVigo I+i Research Group, Galicia Sur Health Research Institute (IIS Galicia Sur), SERGAS-UVIGO, 36312 Vigo, Spain; mar.mosteiro.anon@sergas.es (M.M.-A.); maria.luisa.torres.duran@sergas.es (M.T.-D.); jose.alberto.fernandez.villar@sergas.es (A.F.-V.); 3 School of Industrial Engineering, University of Vigo, 36310 Vigo, Spain; 4 Department of Design in Engineering, University of Vigo, 36208 Vigo, Spain; 5 Centro de Investigación Biomédica en Red, CIBERES ISCIII, 28029 Madrid, Spain; 6 Diagnostic Imaging Department, Hospital Ribera Povisa, 36211 Vigo, Spain; alberto.fernandez.garcia@outlook.es; 7 Pulmonary Department, Hospital Álvaro Cunqueiro, 36312 Vigo, Spain

**Keywords:** artificial intelligence, simulation, healthcare process, hospital management, waiting time, patient flow

## Abstract

**Highlights:**

**What are the main findings?**

- Integrating artificial intelligence (AI) and simulation improves healthcare management, optimizes resource allocation, and reduces waiting times, especially in emergency departments and clinical pathways.
- Most studies rely on supervised learning and discrete event simulation methods, but hybrid AI-simulation strategies remain underutilized, revealing an untapped potential for broader adoption in healthcare processes.

**What is the implication of the main finding?**

- The combined use of AI and simulation can transform healthcare services, enabling more efficient patient flow, improved quality of care, and robust scenario analysis for operational decision-making.
- To fully realize these benefits, future research should focus on expanding applications to new hospital departments, overcoming interoperability and data integration challenges, and advancing toward real-time, adaptable digital twins in healthcare organizations.

**Abstract:**

Background: Healthcare systems face significant challenges due to waiting times, resource shortages, and increasing demand for services. The combination of Artificial Intelligence (AI) and simulation is emerging as a promising solution to optimise healthcare processes, although their joint application remains limited. This systematic review analyses current methodological approaches that integrate both technologies to enhance healthcare management. Methods: A systematic search was conducted in PubMed and IEEE Xplore for articles published between 2014 and 2025, following PRISMA guidelines. The search strategy included terms related to AI, simulation, and healthcare management, and was supplemented by a “snowball” search. Original studies describing combined applications of AI and simulation in healthcare processes were included. Results: Out of 2506 records identified, 22 studies were selected for final analysis, most of which were published between 2021 and 2025, indicating growing interest in the field. The studies show that integrating AI and simulation has the potential to improve the efficiency of healthcare management, optimise resource allocation, and reduce waiting times, particularly in areas such as emergency departments and clinical pathways. Supervised learning algorithms, discrete event simulation (DES), and agent-based systems (ABS) were the most commonly used approaches. Conclusions: The combination of AI and simulation is an emerging field with great potential to revolutionise the management of healthcare processes. However, effective implementation requires overcoming technological, standardisation, and data integration barriers, as well as expanding its application to more hospital departments to maximise its impact.

## 1. Introduction

Currently, one of the main challenges faced by healthcare systems is addressing the long waiting times patients must endure to access specialist consultations and surgical procedures [1,2,3]. This issue is partly attributable to an increasingly ageing and comorbid population, as well as a shortage of healthcare professionals [4]. Furthermore, the situation has been exacerbated by the COVID-19 pandemic [5,6,7]. This reality generates concern among both patients and healthcare staff [8,9], as it negatively impacts the quality of care, increases professional burnout, and contributes to a negative perception of the healthcare system. Altogether, these factors hinder the effective delivery of healthcare services, particularly in such a sensitive domain as health, affecting all parties involved.

To address this issue, one of the most relevant strategic approaches is the optimization of the organization and management of healthcare resources [10]. Careful planning and coordination of healthcare activities and processes are essential for health services to operate efficiently and effectively. In this context, and leveraging the increasing volume of patient data alongside advances in new technologies, there is an opportunity to develop engineering-based approaches aimed at tackling this challenge.

Artificial Intelligence (AI) has already demonstrated remarkable success in diagnostic and predictive applications across multiple medical specialties, such as radiology [11,12,13], pathology [14,15,16], cardiology [17,18,19], and respiratory medicine [20,21,22,23,24,25,26,27]. Machine learning (ML) and deep learning algorithms have achieved expert-level performance in image interpretation, disease classification, and risk prediction, contributing to earlier diagnosis and more personalized treatment strategies [28,29,30,31,32]. These advances illustrate the transformative potential of AI in healthcare, paving the way for its integration into broader management and operational domains.

Among these solutions, the digitalization of healthcare processes stands out for its potential to accurately model the functioning of the healthcare system and provide tools that support informed decision-making, based on reliable predictions and scenario analysis [33]. This digital transformation contributes to enhancing efficiency, reducing time and costs, and improving the quality of care—particularly important in such a complex environment as healthcare [34].

In this regard, simulation models may be the most suitable modelling technique for this purpose, as they have proven to be effective tools for analysing and improving the performance of logistical processes in sectors outside healthcare. They have been successfully employed in areas such as manufacturing, transportation, and warehousing [35]. An illustrative example is the work [36], in which they developed a discrete event simulation (DES) model of a logistical process, aimed at more accurately managing the synchronization of components on an automotive assembly line. In general, simulation models are parameterized representations of real-world systems that enable the computational resolution of problems too complex to be addressed analytically. Although these techniques have traditionally been used in healthcare to model patient anatomical objects or structures [37,38], in recent years their application has expanded significantly to the study and optimization of healthcare processes [39].

The main advantage of having a simulation model of healthcare systems lies in its ability to predict events and trends, as well as to describe processes and activities using quantitative indicators that support decision-making. For example, these models can be used to optimize the allocation of hospital resources [40], define new patient flow pathways [41], or redesign hospital timetables and appointment scheduling [42]. Unlike purely analytical approaches, simulation enables the evaluation of various hypothetical “what-if” scenarios through minor adjustments to the model, without requiring changes to its overall structure. Furthermore, the comprehensibility and interpretability of these models facilitate communication between healthcare professionals and managers, thus supporting the implementation of evidence-based measures. Ultimately, such models can even be integrated with hospital information systems in real time, leading to what is known as a digital twin [43]: a virtual replica of the real process that is automatically updated with system data and enables continuous simulation and adjustment of care delivery. This capability could radically transform organizational processes and revolutionize healthcare management.

However, given the inherent complexity of healthcare processes, constructing a simulation model that is truly reliable and interpretable is time-consuming and requires multiple stages of refinement to accurately parameterize the data collected from the studied reality [39]. To overcome this limitation, it is possible to integrate AI approaches within the simulation framework, harnessing the high predictive power of these techniques [44] alongside the high interpretability and clarity offered by simulation methods [45]. In fact, the combination of AI and simulation is particularly useful in contexts where a single strategy is insufficient to manage the high dimensionality of data, or when the system includes both causal and unknown relationships. In this regard, AI can be used to detect hidden patterns in the data and enhance the predictive capacity of the simulation, while simulation can provide additional or complementary information to enrich AI models. Together, hybrid solutions enable the generation of synthetic data to improve model training, the integration of diverse information sources, and the development of approximate, lower-cost models—thus opening up new possibilities for the optimisation and advanced management of healthcare processes [46].

**Figure 1:**
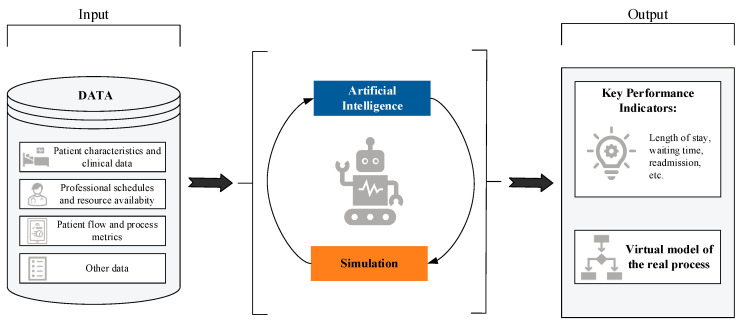
Schematic conceptualization of the topic addressed in this systematic review: the combination of AI models and simulation for the optimization of healthcare processes.

Although both AI and simulation have been extensively studied independently within the healthcare domain [47,48,49,50,51,52]—whether to anticipate clinical complications and thereby improve decision-making, or to streamline resource management in high-pressure situations such as emergency care and reduce hospital stays—there is still a limited number of studies that jointly address both tools within a hybrid approach. The combination of simulation with AI techniques remains a relatively unexplored area, particularly in terms of improving healthcare processes from an operational and planning perspective. Figure 1 provides a schematic illustration of this concept of combining AI with simulation to enhance healthcare processes.

For all the reasons outlined above, the objective of this work is to conduct a systematic review of studies that address the main challenges in healthcare management through innovative methodological approaches combining AI and simulation techniques. The decision to focus the review exclusively on the field of care management is deliberate, driven by the interest in defining a specific area within biomedical engineering where this combination of tools could have a particularly significant impact.

To the best of our knowledge, this review represents a significant contribution to the fields of healthcare and biomedical engineering by providing an updated and expanded perspective on the combination of AI and simulation techniques applied to the management of healthcare processes. Unlike previous works, such as the review by [53], which covered the period 2012–2021 and was specifically limited to the combination of machine learning and simulation, our study spans research published between 2014 and 2025 and broadens the scope to include all types of AI approaches, not just ML. This allows the inclusion of more recent studies and the identification of emerging trends in the field. Furthermore, a more comprehensive search strategy has been defined, incorporating a wider range of key terms related to planning, scheduling, and various modelling approaches. It is also worth noting that, although other recent reviews have focused on related topics—such as the review by [54], which explores the use of ML and optimization techniques in the surgical domain of arthroplasty—this work differs by addressing a broader spectrum of clinical applications and healthcare processes, without being restricted to a specific pathology or specialty.

The structure of this work is as follows: first, the search strategy and study selection process are described in detail; next, the main characteristics of the selected studies are presented and analysed; and finally, a critical analysis of the findings is provided, along with conclusions and potential future research directions that may contribute to progress in this field.

## 2. Methods

The systematic literature review was conducted following the PRISMA guidelines (Preferred Reporting Items for Systematic Reviews and Meta-Analyses), a widely recognized standardized methodology for performing reviews and meta-analyses [55,56]. Specifically, a structured search of articles was carried out using a selected set of key terms and predefined inclusion and exclusion criteria. The use of specific and systematic search strategies enables the identification of research niches and the selection of a limited set of documents that accurately and meaningfully represent the topic under study. Furthermore, the protocol for this systematic review is registered in the international PROSPERO database under identification number CRD420251060727, ensuring traceability and transparency of the methodological process.

### 2.1. Research Question

What are the current methodological approaches, tools and benefits of combining AI and Simulation techniques for optimizing healthcare processes and improving hospital management?

### 2.2. Eligibility Criteria

The systematic literature review was conducted with the aim of describing and analysing applications and use cases where combined approaches of AI and simulation are adopted to develop models applied to management of healthcare processes. These models may serve, for example, to support clinical decision-making or to design personalized care pathways that allow better management of patients and healthcare professionals.

The inclusion criteria were as follows:

1. Original peer-reviewed research articles. Abstracts, conference proceedings, letters, comments, editorials, and review articles were excluded.
2. Articles written in English, to ensure linguistic consistency among the included studies.
3. Studies focused on applications of combined AI and simulation approaches within the scope of management of healthcare processes.
4. Works providing clear and detailed descriptions of the AI and simulation models employed.

### 2.3. Information Sources

In this study, the search was conducted on 28 May 2025, exploring two databases: PubMed and IEEE Xplore. PubMed was selected for its specialization in health sciences and biomedicine, ensuring comprehensive access to clinical studies and medical applications. IEEE Xplore was chosen as a reference source for engineering, informatics, and emerging technologies, where advances in computational simulation and AI are published. Both databases are widely recognized as among the most relevant in the fields of scientific healthcare and engineering research, are constantly updated, and comprise a large volume of relevant publications. Additionally, a complementary “snowball” search was performed through citation chasing, which involved reviewing the reference lists of the selected articles and using Google Scholar to identify additional peer-reviewed studies citing those works. All studies retrieved through this method were subjected to the same inclusion and exclusion criteria, ensuring that only peer-reviewed research articles meeting our eligibility requirements were included, with the aim of maximizing the thoroughness of the review and minimizing the risk of omitting relevant studies.

Publications from 2014 to 2025 were included, focusing on recent and established studies, with 2014 chosen as the starting point, coinciding with the emergence of AI in the healthcare sector.

### 2.4. Search Strategy

The general query used to perform the search is detailed in Table 1.

For each database consulted (PubMed and IEEE Xplore), the search was adapted to the specific syntax and operators of each platform.

PubMed:

((intelligen* [Title/Abstract]) OR (“machine learning” [Title/Abstract]) OR (“deep learning” [Title/Abstract]) OR (“reinforcement learning” [Title/Abstract]) OR (“expert system” [Title/Abstract]) OR (“decision support system” [Title/Abstract]) OR (“knowledge-based system” [Title/Abstract]))

AND

((simulation [Title/Abstract]) OR (“digital twin” [Title/Abstract]) OR (“agent-based” [Title/Abstract]) OR (“discrete-event” [Title/Abstract]) OR (“markov chain” [Title/Abstract]))

AND

((healthcare [Title/Abstract]) OR (“health care” [Title/Abstract]) OR (“health system” [Title/Abstract]) OR (“health services” [Title/Abstract]) OR (“patient flow” [Title/Abstract]) OR (“clinical management” [Title/Abstract]) OR (“clinical process” [Title/Abstract]) OR (“hospital management” [Title/Abstract]) OR (“hospital process” [Title/Abstract]) OR (“workflow” [Title/Abstract]) OR (“waiting time” [Title/Abstract]))

IEEE Xplore:

(“intelligent*” OR “machine learning” OR “deep learning” OR “reinforcement learning” OR “expert system” OR “decision support system” OR “knowledge-based system”)

AND

(“simulation” OR “digital twin” OR “agent-based” OR “discrete-event” OR “Markov chain”)

AND

(“healthcare” OR “health care” OR “health system” OR “health services” OR “patient flow” OR “clinical management” OR “clinical process” OR “hospital management” OR “hospital process” OR “workflow” OR “waiting time”)

The development process of the search strategy was based on three key concepts: AI, simulation, and healthcare management. For each concept, search terms were selected based on their relevance and direct relation to the concept, complemented by synonyms and terms identified in the titles, abstracts, and subject indexes of records in preliminary searches. An initial draft of the search strategy was prepared and refined by incorporating new terms identified from key articles and previous reviews on the same topic. Specifically, some terms were incorporated based on the results obtained from a review by [53], allowing the experience and previous findings in this area to be leveraged. However, unlike the review by [53], this review expanded the range of terms used, enabling a more comprehensive coverage of the current literature.

After applying the search query, a temporal filter was applied to restrict results to articles published between 1 January 2014 and 28 May 2025. In PubMed, this was implemented using the publication date filter, and all resulting articles were reviewed without applying additional content type restrictions. In IEEE Xplore, the publication year range was set to 2014–2025, and the content type was restricted to “Journals” and “Early Access Articles” only.

### 2.5. Selection Process

The study selection process was carried out in accordance with PRISMA guidelines, using its flow diagram to transparently document each stage of the procedure. First, after searching the selected databases, all results were compiled, and duplicates were removed. A first screening of titles and abstracts was then conducted, excluding articles that did not meet the predefined eligibility criteria. In the next phase, a full-text review was performed on the studies selected in the previous step, in order to examine their content in greater depth and exclude those that did not meet more specific eligibility criteria based on the full manuscript. During this second screening, articles for which full-text access was not available were also excluded.

### 2.6. Collection Process and Data Items

The identifying information of the articles was compiled in a customized Microsoft Excel spreadsheet created by the authors, with the aim of recording the following relevant data for each study under specific fields:

1. ID: sequential number to identify the reviewed studies.
2. Authors: names and surnames of the article’s authors.
3. Title: full title of the article.
4. Digital Object Identifier (DOI): unique identifier of the publication.
5. Abstract: full abstract of the article.
6. Year: year of publication.
7. Application area: description of the healthcare domain or specific problem addressed.
8. Study objective: main purpose (e.g., resource optimization, prediction, planning, etc.).
9. Combined AI–simulation approach: description of the strategy used to integrate AI and simulation.
10. AI algorithms: type of AI algorithms employed (e.g., Random Forest (RF), Neural Network (NN), etc.).
11. Programming environment: computational tools used to implement the AI algorithms (e.g., Python, MATLAB, etc.).
12. AI rationale: justification for the use of the AI approach.
13. Simulation models: simulation technique applied (e.g., discrete-event simulation (DES), agent-based simulation (ABS), etc.).
14. Simulation tools: computational tools used to implement the simulation models (e.g., Arena, AnyLogic, etc.).
15. Simulation rationale: justification for the use of the simulation model employed.
16. Benefits of applying AI–Simulation: specific advantages reported from combining both techniques.

Data collection was carried out exclusively based on the information available in the articles themselves, without the need to contact the authors for additional information.

### 2.7. Risk of Bias

All collected information was recorded in an Excel file, structured into pre-agreed fields and shared among all reviewers. Each full-text article was independently reviewed by two reviewers (J.A.-V. and M.C.-G.) to minimize bias and misinterpretation. Discrepancies in inclusion/exclusion decisions were resolved through consensus, with a third reviewer (A.F.-V.) consulted when needed.

To assess methodological quality and risk of bias, we employed the Mixed Methods Appraisal Tool (MMAT) [57,58], a validated instrument for evaluating heterogeneous research methodologies. Given the diverse methodological approaches in this review, the MMAT was the most appropriate tool to provide consistent quality assessment across different study designs.

Each study was independently rated by two reviewers using the MMAT’s seven questions divided into two stages. The first stage consisted of two screening questions to ensure that the included studies were empirical research:

1. Are there clear research questions?
2. Do the collected data allow addressing the research questions?

The second stage comprised five descriptive criteria questions specific to quantitative descriptive studies:

1. Is the sampling strategy relevant to address the research question?
2. Is the sample representative of the target population?
3. Are the measurements appropriate?
4. Is the risk of nonresponse bias low?
5. Is the statistical analysis appropriate to answer the research question?

Ratings were categorized as “Yes”, “No” or “Can’t tell”, and disagreements were resolved through consensus or third-reviewer arbitration.

### 2.8. Effect Measures

No specific effect measures were employed in this systematic review, as no quantitative synthesis or meta-analysis of the results from the included studies was conducted. The extracted information is presented descriptively, focusing on the main characteristics, applications, and outcomes of each study. This decision was made due to the considerable heterogeneity among the selected studies in terms of methodological design, study populations, evaluated interventions, and measured outcomes, which prevented direct comparison and valid statistical aggregation.

### 2.9. Synthesis Method

The collected data were examined and analysed with the aim of:

1. Describing temporal research trends and the characteristics of the included studies in relation to the combined use of AI and simulation in healthcare processes.
2. Illustrating the main application areas of combined AI and simulation approaches in healthcare processes.
3. Presenting the most relevant strategies for effectively combining AI and simulation.
4. Discussing the tools and platforms used to implement the proposed approaches.

The analysis was graphically represented using MS Excel, through line and pie charts, to optimally display the distribution of the collected data. In addition, a systematic synthesis of the studies included at the final stage of selection was provided, categorizing the analysed works according to the main application areas identified during the process, as well as the individual and combined methodological approaches applied.

## 3. Results

### 3.1. Study Selection

**Figure 2:**
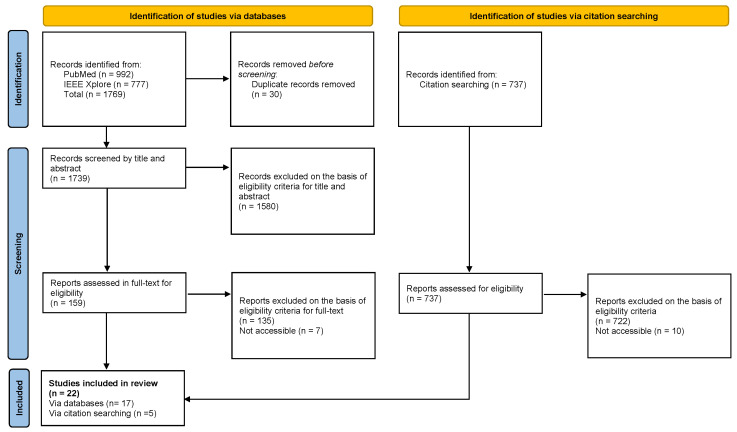
PRISMA Flow Diagram.

The flow diagram of the selection process, in accordance with PRISMA guidelines, is shown in Figure 2. As indicated in the diagram, the systematic search strategy comprised two components: database searching in PubMed and IEEE Xplore, which yielded 1769 studies, and snowball searching through citation tracking and reference list screening, which identified an additional 737 records. In total, 2506 records were identified across both search strategies.

From the 1769 database records, duplicates were removed and titles and abstracts were screened, resulting in 159 studies selected for full-text assessment. During the full-text review, 7 studies were excluded due to lack of access, and the remaining 152 were assessed against the inclusion criteria, of which 17 met the requirements.

Subsequently, from the 737 records identified through documents that cited any of the initially included studies, as well as the reference lists of those studies. An additional 5 studies that met the inclusion criteria were incorporated. In total, 22 articles were included in the systematic review.

During the selection process, numerous articles from the original list obtained from the two explored databases were excluded. The main reasons for exclusion include:

1. The absence of a combined AI and simulation approach. For example, studies focusing solely on AI [48,49,59,60] or solely on simulation [61,62,63,64].
2. Articles centred on the design and development of AI and simulation models without a clear application in the context of healthcare processes. For instance, the work by [65] presents a technical strategy as a proof of concept, but without a specific application to healthcare processes.
3. Studies that do not provide sufficient information on the AI models [66,67,68,69] or on the simulation models [70,71,72,73] used.
4. Studies focusing on non-hospital contexts, such as medical transport [74], disease spread at the population level [75,76,77,78], or modelling of regions outside the clinical environment [79,80,81,82], as the focus of this review is on the internal management of healthcare processes.

### 3.2. Study Characteristics

Table 2 presents the 22 articles included in this review, along with their main characteristics. The table includes the following fields: Authors, Year of Publication, Field of Application, Combined AI–Simulation Approach, AI Algorithm, AI Tool, Simulation Model, and Simulation Tool.

### 3.3. Methodological Quality Assessment

**Figure 3:**
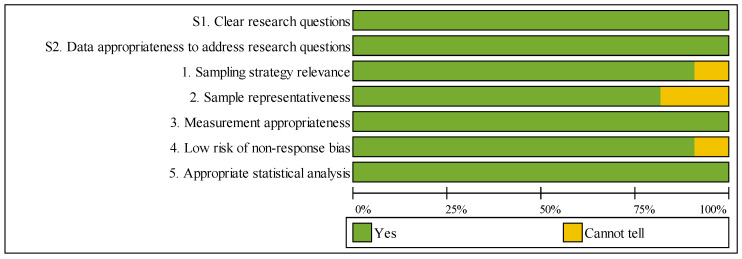
Summary of MMAT Methodological Quality Assessment.

All 22 included studies were evaluated using the MMAT. Detailed individual quality scores for each study are presented in Table 3, with a visual summary of results shown in Figure 3. Of these, 18 studies achieved maximum quality scores (100%), 1 study scored 85.7%, 2 studies scored 71.4% and 1 study scored 57.1% (proof-of-concept with acknowledged limitations).

Both screening questions demonstrated 100% compliance across all studies, confirming clear research questions and appropriate data. Regarding the five descriptive criteria, assessment results were as follows: sampling strategy relevance (90.9% “Yes”), sample representativeness (81.8% “Yes”), measurement appropriateness (100% “Yes”), low nonresponse bias risk (90.9% “Yes”), and appropriate statistical analysis (100% “Yes”). Four studies received “Cannot tell” ratings for sample representativeness due to single-institution designs, though all maintained methodological rigor within their specific contexts.

The high overall quality reflects the review’s rigorous inclusion criteria and demonstrates the robustness of the included evidence across heterogeneous methodologies.

### 3.4. Results of Individual Studies

#### 3.4.1. Research Trends

**Figure 4:**
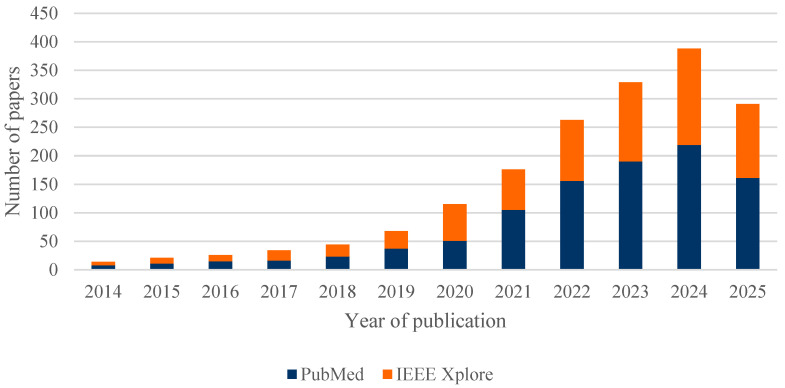
Number of papers published between 2014 and 2025, separated by the two databases: PubMed and IEEE Xplore.

Figure 4 illustrates the evolution of scientific output on the combination of AI and simulation in healthcare processes over the period 2014–2025, based on the two main databases: PubMed and IEEE Xplore. A steady and notably accelerated growth can be observed in recent years, reflecting the increasing interest and consolidation of these technologies within the healthcare sector.

It is worth noting that the data for 2025 only reflect the first months of the year, so the annual figure is not yet complete. Of the total 1769 articles analysed, 82% (1447) were published between 2021 and 2025, highlighting the recent acceleration of research in this field.

**Figure 5:**
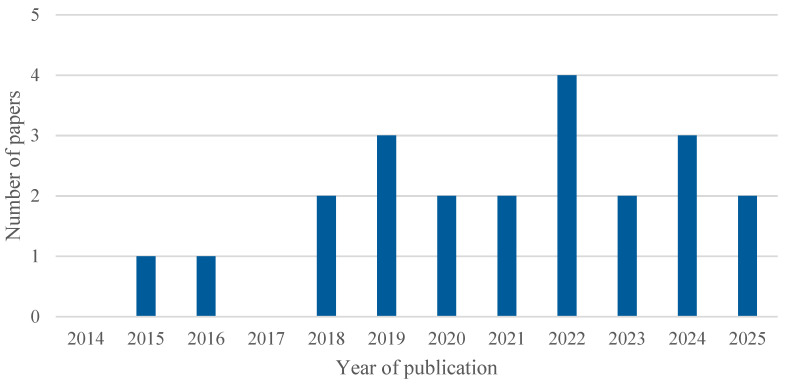
Number of included studies published over time.

The analysis of the 22 articles included in this systematic review, as shown in Figure 5, confirms that the upward trend identified in the overall set of publications is also reflected in the selected studies. However, the limited number of studies ultimately included highlights a specific gap in the literature concerning the combined use of AI and simulation in healthcare processes, underscoring the need to further promote research in this area.

#### 3.4.2. Main Application Scenarios

Among the included studies, three main categories were identified in which combined AI and simulation approaches are applied to healthcare processes:

1. Emergency Department (ED).
2. Clinical Pathways.
3. Other Hospital Departments.

**Figure 6:**
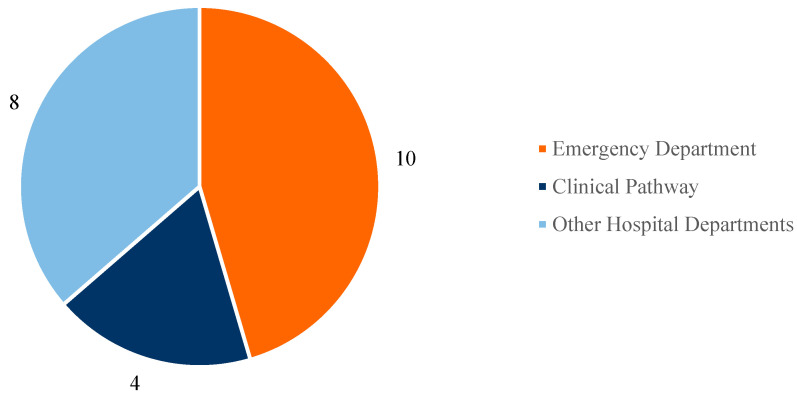
Main application scenarios.

Figure 6 presents a pie chart illustrating the distribution of the included studies according to their application scenario.

Based on the conducted search, most of the selected studies focus on Emergency Department (ED) management (a total of 10 articles), followed by the analysis of clinical pathways for specific diseases (4 studies), while the remaining studies address other healthcare scenarios in various hospital departments (8 studies).

There is a particularly strong interest in applying optimization strategies within ED settings, driven by several factors. Although EDs do not account for the majority of hospital admissions, they do experience a high volume of entries, characterized by significant variability and non-stationarity (e.g., seasonal influences, trends), as well as the diversity and complexity of cases treated. These conditions create bottlenecks in operational flows and hinder efficient prioritization and resource management, exposing EDs to saturation scenarios that impact both staff workload and patients’ perceived quality of care. This context highlights the need for solutions aimed at optimizing resources, reducing waiting times, and improving patient flow. In this regard, advanced technologies such as AI have shown great potential for enhancing ED operational performance due to their strong predictive capabilities. These tools have been used, for instance, to predict patient inflow to EDs [98] and to optimize resource allocation [100].

Several other studies focus on the analysis of clinical pathways—i.e., the diagnostic and/or therapeutic journey of specific conditions, such as acute coronary syndrome [92] or intravenous thrombolysis [84]. These types of studies cannot be assigned to a single hospital department, as they span multiple units and stages of the healthcare process, and are therefore classified under the clinical pathway category.

Lastly, other studies investigate specific hospital departments such as radiology [86] or oncology [89]. It is worth noting that the number of publications addressing departments other than EDs is increasing, indicating a gradual expansion of AI–simulation integration into new areas of healthcare. Nonetheless, there are still hospital departments where such solutions have not yet been developed, revealing opportunities for future research.

#### 3.4.3. Strategies for Combining AI and Simulation

The strategies adopted to integrate AI and simulation in healthcare processes can be classified into four main categories:

1. AI-driven Simulation.
2. Simulation-driven AI.
3. Hybrid AI-simulation.
4. Reinforcement Learning.

**Figure 7:**
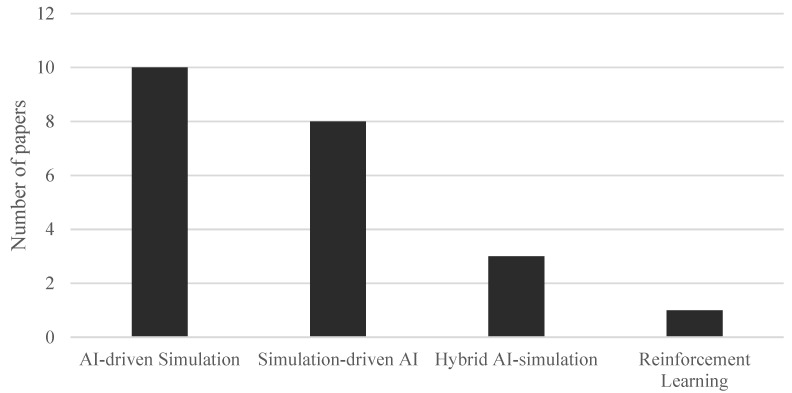
Strategies to combine AI and simulation.

This four-category classification is based on the systematic review by [53]; however, in the present work, minor adjustments have been made to the definitions to better reflect the strategies observed in the articles included in this review. Figure 7 shows the distribution of these approaches across the reviewed literature.

In the first case—AI-driven Simulation—the primary focus is on simulation, with AI integrated to support optimization processes by providing surrogate input data to the numerical model. This integration is particularly useful for overcoming one of the main limitations of traditional simulation approaches: their limited ability to capture non-stationary trends in data, such as fluctuations in patient flow due to seasonality or changes in healthcare demand (e.g., the probability of ICU admission [99]). Moreover, patient flow simulation models have been criticized for often ignoring case heterogeneity, which limits their contribution to personalized medicine and value-based healthcare [83].

This category also includes studies in which AI is used to optimize the results obtained from simulation. A relevant example is the use of GA, which have proven especially useful for fine-tuning parameters and enhancing simulation performance in complex healthcare scenarios [102].

An emerging approach within AI-driven Simulation involves the use of advanced natural language processing technologies—particularly LLMs—to automate the creation of simulation models from textual descriptions. According to study by [97], it has been shown that an LLM such as GPT-4o can automatically generate DES models with accuracy comparable to traditional software, without requiring extensive expert knowledge in simulation.

As shown in Figure 7, this is the most widely used approach for combining AI and simulation among the reviewed studies.

In the second case—Simulation-driven AI—the central role is played by AI, while simulation serves as an additional source of information for AI algorithms by generating synthetic training data in a controlled environment. This strategy is particularly valuable for addressing one of AI’s key limitations: its potential unreliability when real datasets are small, incomplete, or heterogeneous. In healthcare, data often exhibit high variability, errors, or missing information in electronic health records, which hampers the proper training of AI models and demands effective data management and enrichment strategies [85].

This category also includes studies where simulation is used to validate the outputs of predictive AI algorithms. An example is the work by [86], in which simulation was used to quantify the impact of implementing a CNN-based intelligent system for prioritizing radiological exams within the clinical workflow.

In the third case—Hybrid AI–Simulation—AI algorithms are embedded within the simulation model to detect specific patterns in the data or to identify simpler, more efficient submodels. This strategy helps overcome the limitations of both AI—which often lacks full understanding of the healthcare process—and simulation, which may struggle to accurately replicate complex or non-stationary subphenomena. Hybrid models thus combine data-driven surrogates, which are useful for modelling sub-processes with unknown dynamics thanks to the predictive power of AI, with high-fidelity simulations applied to well-understood sub-processes governed by known laws.

A practical example of this approach can be found in the study by [89]. In this work, a clustering algorithm is used to group patients according to their clinical characteristics and treatment needs. These groups are then incorporated into a simulation model representing patient flow and the available resources in a chemotherapy unit. Subsequently, optimisation techniques are applied to adjust appointment planning and scheduling, enabling more efficient resource allocation and reduced waiting times. In this way, AI supports demand segmentation and forecasting, while simulation assesses the operational impact of different planning strategies—combining the strengths of both approaches to address a complex healthcare management problem.

Finally, a distinct category is represented by Reinforcement Learning, in which intelligent agents interact with a simulated environment to continuously adapt and autonomously learn optimal behaviour rules based on the rewards received, whether positive or negative, dynamically adjusting to the context [94]. It is worth noting that only one study included in this review specifically focuses on Reinforcement Learning, highlighting that this line of research remains an emerging niche within the field of healthcare processes.

#### 3.4.4. Programming Environments and Algorithms

**Figure 8:**
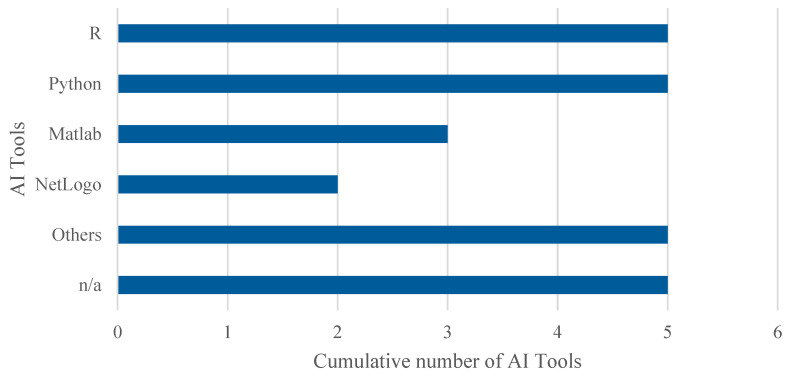
Cumulative number of adopted tools for AI. The overall number of occurrences is higher than the total number of included studies as some works adopted more than one tool to implement AI algorithms.

Regarding the tools used to implement AI algorithms, a detailed analysis of the articles included in this systematic review reveals, as shown in Figure 8, the following distribution. Note that percentages exceed 100% because several studies employed multiple tools in combination. Thus, 22.7% (5 out of 22) employed R, using libraries such as caret, epiR, randomForest, and ROCR. An equal percentage opted for Python, with several studies highlighting the use of the Scikit-learn library. MATLAB was used in 13.6% (3 out of 22) of the cases, while 9.1% (2 out of 22) utilised NetLogo along with the BehaviorSpace module to apply GA in ABS models. The remaining 45.5% (10 out of 22) did not specify the platform used or employed less common tools such as SAS, Stata, Java, GPT-4o, Prophet, and Orange.

**Figure 9:**
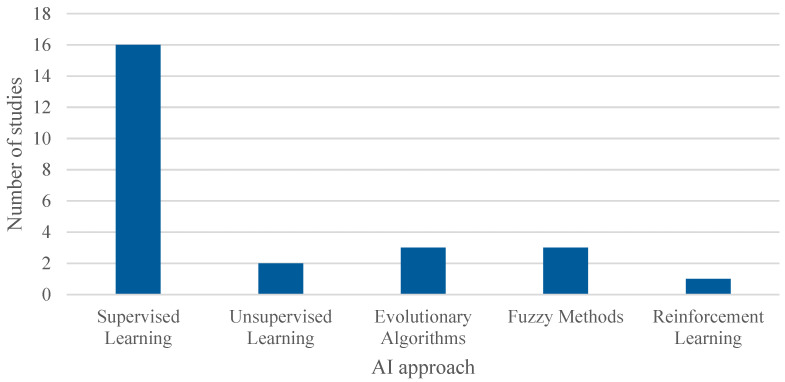
Cumulative number of adopted AI algorithms. The overall number of occurrences is higher than the total number of included studies as some works adopted more than one algorithm to compare the performances of the models.

On the other hand, a wide variety of AI algorithms were used across the reviewed articles, as shown in Table 4 and illustrated in Figure 9. To facilitate analysis, these algorithms were grouped into five main categories (note: the number of studies exceeds 22 as several studies employed multiple algorithms): Supervised Learning, which was the most commonly employed category in the studies; Unsupervised Learning; Evolutionary or Metaheuristic Algorithms, including GA; Fuzzy Methods; and Reinforcement Learning.

This classification provides a clearer understanding of the predominant AI techniques used in combination with simulation within the context of healthcare process management.

**Figure 10:**
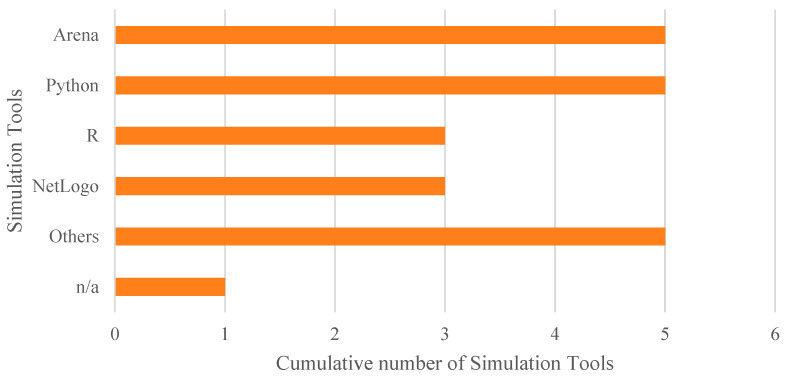
Cumulative number of adopted tools for simulation.

Regarding simulation models, a notable diversity of tools was observed. As shown in Figure 10, 22.7% of the studies (5 out of 22) used Arena and Python (with libraries such as SimPy and NumPy), respectively, for simulation, while 13.6% (3 out of 22) employed R (with the simmer library) and NetLogo, the latter mainly for ABS models. The remaining 27.3% (6 out of 22) used other tools such as Java, FlexSim, AnyLogic, and Enterprise Dynamics, or did not specify the platform used.

Although Python and R stand out as widely used tools for implementing the proposed models, commercial solutions like Arena and NetLogo are also frequently employed. Additionally, some studies have developed customized solutions—for example, in the study by [87], where the advantage of using tailor-made, easily adjustable tools is emphasized, as they allow the construction of more flexible models adapted to the specific needs of each organization.

**Figure 11:**
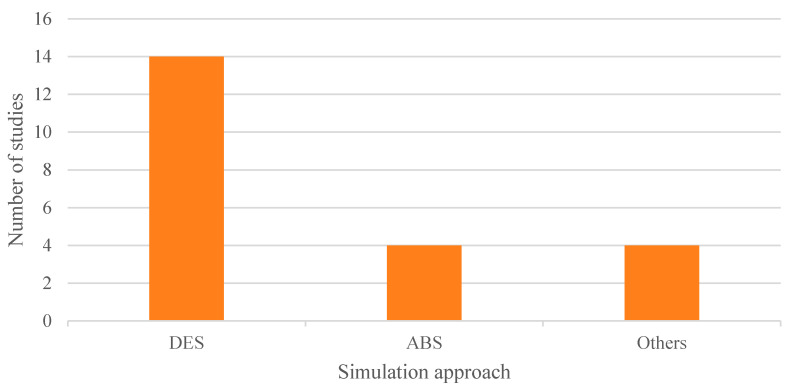
Adopted simulation approaches.

Finally, regarding the type of simulation approach implemented, two main methods were identified: DES, which was by far the most commonly used in the articles included in the review, and ABS, as shown in Figure 11. In addition, some studies employed other more specific techniques tailored to particular problems, such as discrete distributions, Monte Carlo simulation, and Markov chains.

### 3.5. Results of Syntheses

To provide the reader with a clearer understanding of the applications identified in this review and the individual and combined methodological approaches applied, Table 5 presents a schematic summary of each analysed article.

## 4. Discussion

### 4.1. Current State of Research

This systematic review is one of the few to thoroughly examine the state of the art in research combining AI and simulation in the management of healthcare processes. It has been found that there is still considerable scope for further development in the effective implementation of these methodologies. In fact, studies that fully exploit the combined potential of simulation and AI remain limited—only 22 studies were ultimately included after the selection process. In our view, there is a clear need to move towards broader application of hybrid approaches that integrate AI and simulation to meaningfully improve the management of healthcare services.

The benefits of combining both methodologies are numerous, particularly the ability to model complex processes and generate more accurate and context-specific outcomes. However, this integration poses significant technical and organisational challenges, as it requires the use of multiple tools that must be properly interconnected, thereby increasing implementation complexity. While some commercial simulation platforms, such as NetLogo with its *BehaviourSpace* module, already incorporate AI algorithms, this field is still in its early stages. Furthermore, the lack of standardisation in model implementation hinders widespread adoption and limits both interoperability and reproducibility.

Despite these challenges, the robustness of current research provides a solid foundation upon which the field is advancing.

### 4.2. Methodological Considerations

The consistently high quality of included studies indicates that AI-simulation research in healthcare has achieved substantial methodological maturity. However, this uniformity raises important concerns: publication bias likely favours rigorous, positive studies, potentially excluding inconclusive or negative findings. Additionally, the predominance of well-resourced academic institutions in this field suggests that methodological excellence may not be uniformly accessible across different healthcare systems.

The variation in sample representativeness—lower scores for single-institution studies—highlights a critical tension: rigorous case studies provide essential feasibility evidence but offer limited generalizability across diverse healthcare contexts. This limitation becomes particularly acute given international differences in healthcare systems and resources.

The demonstrated methodological rigor validates the credibility of findings across the included studies and enables the field to move beyond proof-of-concept toward more diverse, real-world applications.

### 4.3. Applications in Hospital Departments

The breadth and heterogeneity of applications reviewed in this study exemplifies how the methodologically sound research base is translating into diverse practice areas. The potential impact of research combining AI and simulation is particularly significant in hospital management, with special attention given to ED. Resource optimisation is a critical aspect in any healthcare facility, and the simulation of various scenarios—such as operating theatres, intensive care unit beds, general ward capacity, staffing levels, medications, and medical supplies—enables healthcare managers to estimate care demand under different conditions, analyse the impact of bottlenecks, and test possible solutions before implementation. This helps reduce the risk of decision-making errors and supports more efficient management that is better aligned with the realities of the hospital setting.

In the case of studies focused on clinical pathways, considerable heterogeneity was observed. While all share the common aim of improving healthcare management through the joint application of AI and simulation, their specific approaches and objectives vary greatly, reflecting the complexity and diversity of healthcare processes that span multiple hospital departments.

On the other hand, some studies apply AI and simulation in other hospital departments such as oncology, radiology, or operating theatres. This is a significant development, as it shows that these technologies are not limited to emergency settings but are beginning to expand into other key areas of hospital care. However, there are still departments where such engineering solutions have not yet been implemented, reinforcing the notion that the full potential of the combined AI–simulation strategy remains largely untapped. The studies published so far represent a pioneering approach and pave the way for future research and applications in currently unexplored areas.

### 4.4. Computational Tools and Algorithms Employed

Another important aspect concerns the computational tools and algorithms used in the selected studies. Both commercial and open-source platforms were employed to implement the algorithms, with R and Python being the most common open-source options, and MATLAB, NetLogo, and Arena among the commercial tools.

The predominance of certain tools and methodologies identified in this review warrants critical reflection on its implications for the field. Regarding simulation approaches, the overwhelming preference for DES over other paradigms—such as agent-based simulation or system dynamics—may be attributed to its intuitive representation of patient flow and resource utilization, as well as the maturity and widespread availability of DES software platforms. However, this dominance may inadvertently limit the exploration of alternative simulation paradigms that could offer complementary insights into complex, feedback-driven healthcare processes, particularly when modelling emergent behaviours or systemic interactions.

Similarly, the prevalent use of both commercial platforms (such as Arena, MATLAB, and NetLogo) and open-source solutions (such as Python and R) raises important considerations regarding accessibility, cost, and sustainability. While commercial platforms often provide user-friendly interfaces and integrated modules that facilitate model development, they may create financial and technical barriers for healthcare institutions in low-resource settings, potentially limiting the equitable adoption of these advanced methodologies. Conversely, open-source tools promote transparency, reproducibility, and collaborative development, yet may require greater technical expertise and lack the standardised support structures offered by commercial vendors. This duality underscores the need for greater efforts toward developing open, standardised frameworks that balance accessibility with functionality.

Furthermore, the predominance of supervised learning algorithms—identified in the majority of AI implementations—reflects the current focus on predictive accuracy and classification tasks. However, this emphasis may overlook the potential of alternative approaches, such as reinforcement learning, to enable adaptive, autonomous decision-making in dynamic healthcare environments. The scarcity of studies employing reinforcement learning or hybrid AI strategies suggests that methodological and computational barriers remain significant obstacles to exploring the full spectrum of AI capabilities in healthcare management.

### 4.5. Standardisation and Integration Challenges

The lack of standardisation in model implementation—evidenced by the diversity of tools, programming languages, and integration strategies—poses challenges for interoperability, reproducibility, and scalability across different healthcare settings. This fragmentation hinders the ability to compare results across studies, validate findings in different contexts, and transition from research prototypes to operational deployment. Establishing common data formats, standardised model interfaces, and validation protocols would facilitate the broader adoption and real-world impact of combined AI-simulation approaches in healthcare management.

Yet standardisation alone is insufficient to ensure successful translation of these methodologies into clinical practice. Even where technical standards exist, a broader implementation gap persists—one that extends beyond tool compatibility to encompass the fundamental challenge of transitioning from validated research prototypes to sustainable, integrated solutions within the complex realities of healthcare systems.

### 4.6. Translating Research into Clinical Reality: The Sustainability Challenge

A critical examination of the included literature reveals a significant and often underexamined limitation: the predominance of proof-of-concept studies over genuine real-world implementations. Of the 22 included studies, only a minority demonstrate actual deployment in clinical practice, and even fewer report meaningful follow-up data on the operational sustainability and clinical impact of the implemented tools. Most published research represents either theoretical models validated with retrospective data or temporary case studies that do not extend into sustained clinical practice.

This gap between methodological validation and operational implementation reflects a broader challenge in healthcare technology adoption: the difficulty of transitioning from promising research prototypes to sustainable, integrated solutions within complex hospital ecosystems. While validation studies are essential for establishing feasibility, the absence of longitudinal implementation data—tracking tool usage, clinical staff adoption, actual improvements in patient outcomes, and cost-effectiveness over extended periods—substantially limits our understanding of whether these AI and simulation approaches genuinely transform clinical practice or remain confined to research environments.

This implementation gap underscores not only the novelty of the field but also highlights the need for future research to prioritize implementation science frameworks and long-term follow-up studies that assess real-world effectiveness, organizational barriers, and sustainability beyond the initial pilot phase. Without such evidence, stakeholders and healthcare administrators face considerable uncertainty when deciding whether to invest in these technologies for their institutions.

### 4.7. Innovative Advances: From AI-Generated Modelling to Digital Twins

A particularly noteworthy advancement is found in study [97], which introduces the use of LLMs for the automatic generation of DES models from textual descriptions. This study demonstrates that an LLM such as GPT-4o can construct DES models without the direct involvement of a simulation expert, achieving results comparable to those produced by traditional software like Arena. This approach opens a new paradigm by accelerating and simplifying the development of simulation models in healthcare, making it easier and more efficient to optimise resources and analyse complex scenarios.

On the other hand, it is important to highlight the absence of real-world applications of digital twins in the reviewed studies. Although the natural progression following the creation of a simulation model would be to advance towards interconnected systems with the actual process, capable of automatically capturing data and making real-time decisions, such solutions have yet to be implemented in combination with AI in clinical practice. The work [43] proposes a conceptual framework for the development of digital twins within the hospital setting, particularly in operating theatres and recovery areas, but this remains a proof of concept rather than an established application.

Therefore, future studies should focus on two main lines: expanding the application of AI and simulation to new hospital departments, and progressing towards the development of fully functional digital twins. To achieve effective implementation of these advanced solutions, it is essential to demonstrate their applicability across a wide range of scenarios and contexts. Developing AI and simulation models that are flexible and adaptable remains a significant challenge and one of the main barriers to their widespread adoption in public health. Nevertheless, their development and consolidation could radically transform healthcare management, enabling the creation of reliable digital twins that optimise clinical processes in real time.

### 4.8. Limitations of This Review

Several methodological limitations warrant acknowledgement. First, the search strategy itself presented constraints. The search was limited to PubMed and IEEE Xplore databases; the exclusion of widely used sources such as Scopus represents a notable limitation, though this decision reflected time constraints rather than strategic choice. Furthermore, conference proceedings and preprints were excluded a priori. Whilst conference abstracts can represent cutting-edge research in rapidly evolving fields such as AI and healthcare informatics, preliminary exploratory searches indicated that abstracts addressing the combined application of AI and simulation in healthcare management frequently lacked the methodological detail and rigour necessary for comprehensive assessment. Additionally, the search was restricted to English-language publications, which may introduce language bias and potentially exclude relevant research published in other languages or not published in mainstream English-language journals.

Second, formal quality-assessment frameworks were not applied. Given the exploratory and descriptive scope of this systematic review, along with the considerable methodological heterogeneity of the included studies, structured quality-assessment and certainty-of-evidence frameworks such as PROBAST and GRADE were not employed. These tools are designed primarily for reviews evaluating specific interventions or comparisons to inform clinical recommendations, which differs from the landscape-mapping purpose of this review. Instead, to address internal validity concerns, each full-text article was independently reviewed by two evaluators, with discrepancies resolved through discussion and consensus, and a third reviewer available as arbitrator when necessary. While this approach ensured rigorous screening, formal inter-rater agreement statistics (e.g., Cohen’s kappa) were not computed for screening and data extraction decisions, representing a methodological limitation. However, discrepancies between reviewers were infrequent during both title/abstract and full-text screening.

Finally, the search strategy itself could be refined. Some selected search terms did not contribute significant value to the overall strategy. For future reviews, employing tools such as PubReMiner—which enables analysis of word frequency and optimisation of search strategies through frequency tables—would facilitate the identification of relevant terms and improve the efficiency of scientific literature retrieval.

## 5. Conclusions

The combination of AI and simulation represents one of the most promising strategies to address current challenges in healthcare management. The reviewed studies demonstrate that integrating both technologies enables improved efficiency, optimised resource allocation, and reduced waiting times, whilst facilitating the development of more interpretable tools tailored to healthcare professionals’ real needs.

However, a critical gap exists between methodological promise and clinical reality. Most published research remains at the proof-of-concept stage, with limited evidence of sustained implementation in routine practice. The scarcity of long-term follow-up studies documenting operational deployment, staff engagement, and genuine impacts on patient outcomes underscores the urgent need for future research to prioritise implementation science frameworks that evaluate sustainability and cost-effectiveness over extended periods.

Additional barriers persist, including the effective incorporation of digital twins into clinical practice and the need to broaden these approaches to underexplored hospital departments. Yet the transition from promising research prototypes to operationally sustainable solutions remain an unmet challenge.

Success will require not only continued technical development but equally important efforts to establish implementation pathways, standardised frameworks, and long-term evaluation methods. Only through such comprehensive approaches—combining methodological rigour with implementation science—can these technologies realise their transformative potential in advancing towards a more efficient, sustainable, and patient-centred healthcare system.

## Figures and Tables

**Table 1:** Search strategy.

Concept	Terms	
AI	Intelligent and its derivatives, Machine Learning, Deep Learning, Reinforcement Learning, Expert system, Decision support system, Knowledge-based system	Combine all terms with OR
AND		
Simulation	Simulation, Digital Twin, Agent-based, Discrete-event, Markov chain	Combine all terms with OR
AND		
Healthcare	Healthcare, Health system, Health service, Patient flow, Clinical management, Clinical process, Hospital management, Hospital process, Workflow, Waiting time	Combine all terms with OR

**Table 2:** Study Characteristics.

Authors (Year)	Field of Application	Combined AI-Simulation Approach	AI Algorithms	AI Tools	Simulation Models	Simulation Tools
Abuhay et al. (2023) [83]	Clinical pathway	AI-driven Simulation	XGBoost	Python (SciKit Learn)	Discrete Event Simulation (DES)	Python (SimPy)
Allen et al. (2022) [84]	Clinical pathway	AI-driven Simulation	Logistic Regression (LR), Random Forest (RF) and Neural Network (NN)	Python (SciKit Learn)	Monte Carlo	Python (NumPy)
Atalan et al. (2022) [85]	ED	Simulation-driven AI	RF, Gradient Boosting (GB) and AdaBoost	Orange	DES	FlexSim
Baltruschat et al. (2021) [86]	Radiology	Simulation-driven AI	Convolutional Neural Network (CNN)	n/a	Discrete distribution	n/a
De Deken et al. (2025) [87]	Clinical pathway	Hybrid AI-simulation	Hierarchical clustering	R	Markov chain	R
Gartner and Padman (2019) [88]	ED	AI-driven Simulation	Naïve Bayes (NB), Bayesian networks (BN), Decision Tree (DT) and Decision Rules (DR)	n/a	DES	Arena
Hadid et al. (2022) [89]	Oncology	Hybrid AI-simulation	K-means, Hierarchical clustering and Self-Organizing Maps clustering	n/a	DES	AnyLogic
Hosseini_Shokouh et al. (2022) [90]	ED	Simulation-driven AI	NN and Genetic Algorithms (GA)	MATLAB	DES	Enterprise Dynamics
Kim et al. (2021) [91]	ICU	Simulation-driven AI	LR and XGBoost	R (caret)	DES	R (simmer)
Kovalchuk et al. (2018) [92]	Clinical pathway	Hybrid AI-simulation	K-means and DT	Python (SciKit Learn)	DES	Python (SimPy)
Lazarashouri and Najafi (2024) [93]	ED	Simulation-based AI	Fuzzy	n/a	DES	Arena
Lee et al. (2015) [94]	Oncology	Reinforcement Learning	ɛ-greedy strategies, Interval estimation strategies and Boltzmann exploration strategies	MATLAB	DES	MATLAB
Lin et al. (2025) [95]	Radiology	AI-driven Simulation	Deep Learning	Python	DES	Python (SimPy)
Mazumdar et al. (2020) [96]	Oncology	Simulation-driven AI	LR, RF and other (PLAQR)	R, SAS and Stata	Statistical modelling	R
Miski et al. (2024) [97]	Clinic and medical care facility workflows	AI-driven Simulation	Transformer-based large language model (LLM)	GPT-4o	DES	Python (SimPy)
Nas and Konyuncu (2019) [98]	ED	AI-driven Simulation	AdaBoost, LR, Stochastic gradient regression (SG), DT, GB, RF, Support vector machine (SVM), K-nearest neighbours (KNN), Multilayer perceptron (MLP) and other (LSTM)	Python	DES	Arena
Ortiz-Barrios et al. (2023) [99]	ICU	AI-driven Simulation	RF	R (epiR, caret, randomForest and ROCR)	DES	Arena
Ortiz-Barrios et al. (2024) [100]	ED	AI-driven Simulation	RF	R	DES	Arena
Othman et al. (2016) [101]	ED	AI-driven Simulation	Fuzzy	Java	ABS	Java
Sulis et al. (2020) [102]	ED	AI-driven Simulation	GA	NetLogo (BehaviorSpace)	ABS	NetLogo
Yousefi et al. (2018) [103]	ED	Simulation-driven AI	Feedforward neural network (FNN), Recurrent neural network (RNN) and other (Adaptive neuro-fuzzy inference system (ANFIS))/GA	MATLAB/NetLogo (BehaviorSpace)	ABS	NetLogo
Yousefi et al. (2019) [104]	ED	Simulation-driven AI	FNN, RNN and other (ANFIS)	n/a	ABS	NetLogo

**Table 3:** MMAT Quality Assessment Results.

	Methodological Quality Criteria						
	Screening Questions		Quantitative Descriptive				
Authors (Year)	S1. Clear research questions	S2. Data appropriateness to address research questions	1. Sampling strategy relevance	2. Sample representativeness	3. Measurement appropriateness	4. Low risk of non-response bias	5. Appropriate statistical analysis
Abuhay et al. (2023) [83]	Yes	Yes	Yes	Yes	Yes	Yes	Yes
Allen et al. (2022) [84]	Yes	Yes	Yes	Yes	Yes	Yes	Yes
Atalan et al. (2022) [85]	Yes	Yes	Yes	Cannot tell	Yes	Yes	Yes
Baltruschat et al. (2021) [86]	Yes	Yes	Yes	Yes	Yes	Yes	Yes
De Deken et al. (2025) [87]	Yes	Yes	Yes	Yes	Yes	Yes	Yes
Gartner and Padman (2019) [88]	Yes	Yes	Yes	Cannot tell	Yes	Cannot tell	Yes
Hadid et al. (2022) [89]	Yes	Yes	Yes	Yes	Yes	Yes	Yes
Hosseini_Shokouh et al. (2022) [90]	Yes	Yes	Yes	Yes	Yes	Yes	Yes
Kim et al. (2021) [91]	Yes	Yes	Yes	Yes	Yes	Yes	Yes
Kovalchuk et al. (2018) [92]	Yes	Yes	Yes	Yes	Yes	Yes	Yes
Lazarashouri and Najafi (2024) [93]	Yes	Yes	Cannot tell	Cannot tell	Yes	Cannot tell	Yes
Lee et al. (2015) [94]	Yes	Yes	Yes	Yes	Yes	Yes	Yes
Lin et al. (2025) [95]	Yes	Yes	Yes	Yes	Yes	Yes	Yes
Mazumdar et al. (2020) [96]	Yes	Yes	Yes	Yes	Yes	Yes	Yes
Miski et al. (2024) [97]	Yes	Yes	Cannot tell	Cannot tell	Yes	Yes	Yes
Nas and Konyuncu (2019) [98]	Yes	Yes	Yes	Yes	Yes	Yes	Yes
Ortiz-Barrios et al. (2023) [99]	Yes	Yes	Yes	Yes	Yes	Yes	Yes
Ortiz-Barrios et al. (2024) [100]	Yes	Yes	Yes	Yes	Yes	Yes	Yes
Othman et al. (2016) [101]	Yes	Yes	Yes	Yes	Yes	Yes	Yes
Sulis et al. (2020) [102]	Yes	Yes	Yes	Yes	Yes	Yes	Yes
Yousefi et al. (2018) [103]	Yes	Yes	Yes	Yes	Yes	Yes	Yes
Yousefi et al. (2019) [104]	Yes	Yes	Yes	Yes	Yes	Yes	Yes

**Table 4:** Classification of AI algorithms.

Main Category	Included Algorithms
Supervised Learning	LR, DR, DT, RF, SG, SVM, KNN, MLP, PLAQR, GB, AdaBoost, XGBoost, NB, BN, NN, CNN, FNN, RNN, LSTM, Deep Learning and Transformer-based LLM
Unsupervised Learning	K-means, Self-Organizing Maps clustering and Hierarchical clustering
Evolutionary and Metaheuristic Algorithms	GA
Fuzzy Methods	Fuzzy and ANFIS
Reinforcement Learning	ɛ-greedy strategies, Interval estimation strategies and Boltzmann exploration strategies

**Table 5:** Results of Syntheses.

Authors (Year)	Study Objective	Benefits of Using AI	Benefits of Using Simulation	Added Value of Combination
Abuhay et al. (2023) [83]	Improving the accuracy and credibility of Acute Coronary Syndrome patient flow simulation by integrating AI to predict length of stay (LoS)	More accurate, data-driven prediction of LoS, accounting for seasonality, trends, and patient heterogeneity	Captures complex, dynamic patient flows and resource constraints across multiple departments; enables scenario analysis and bottleneck identification	Reduces uncertainty, increases model credibility and acceptance, enables individualized scenario analysis, and improves implementation of simulation-driven solutions compared to traditional stochastic-only models
Allen et al. (2022) [84]	Maximizing benefit and increasing rates of intravenous thrombolysis in acute stroke	Accurately predict the likelihood of a patient receiving thrombolysis, quantifies factors influencing treatment, enables hospital-level benchmarking	Clinical pathway simulation seeks to mimic each patient’s journey through clinical pathway and quantifies the potential improvement in thrombolysis rates	Enables scenario analysis combining patient-level decision prediction with process simulation, supports targeted interventions, predicts combined effects of organizational and clinical changes, facilitates more informed, data-driven improvement strategies
Atalan et al. (2022) [85]	Estimating the number of patients treated and patient waiting time as output variables, considering the number and cost of healthcare resources employed in the ED. Analysing the impact of different cost coefficients on these outcomes and overall hospital costs	Provides fast, accurate estimation of the number of patients treated and waiting time based on resource configurations and cost coefficients, supporting data-driven decision-making	Enables detailed modelling of complex, stochastic ED operations, allowing for scenario analysis and rapid evaluation of changes in resource allocation and cost structures	Integration allows for robust, scenario-based optimization of resource allocation: AI models trained on simulation data can quickly estimate the impact of different resource and cost configurations, supporting evidence-based resource planning and cost-benefit analysis
Baltruschat et al. (2021) [86]	Assessing whether AI-driven smart worklist prioritization can reduce report turnaround times (RTATs) for critical findings in chest X-rays (CXRs) instead of the standard FIFO sorting	Enables real-time, automated triage of CXRs by urgency, significantly reducing average RTATs for critical findings, approaching the theoretical optimum of perfect classification	Allows robust, scenario-based evaluation of workflow changes, quantifies both average and maximum RTATs under realistic clinical conditions	Integration provides evidence that AI-driven prioritization can safely and substantially improve workflow efficiency
De Deken et al. (2025) [87]	Mapping and analysing the dynamic, sequential care pathways of paediatric traumatic brain injury patients, aiming to identify subpopulations and improve understanding of treatment patterns and care variability	Enables us to identify meaningful groups of patients with similar care trajectories, facilitating further analysis and interpretation	Provides a probabilistic, temporal representation of care sequences	Detailed, explainable pathway mapping; improves planning by clustering real-world patient data into interpretable care trajectories
Gartner and Padman (2019) [88]	Understanding and predict under-/correct-/overestimation of waiting time perception in the ED to inform staffing decisions and improve satisfaction	Identification of key factors influencing waiting time perception. Supports targeted interventions to address patient dissatisfaction	Allows for scenario analysis of operational changes (e.g., staffing adjustments) and quantifies their impact on patient perceptions and satisfaction	Integration enables the design and testing of interventions (such as staffing changes) that can reduce the number of patients who overestimate waiting times, potentially increasing satisfaction and operational efficiency
Hadid et al. (2022) [89]	Minimizing makespan and overtime in Outpatient Chemotherapy Appointment	Clustering similar appointments reduces the impact of unpunctual arrivals, improving computational efficiency and schedule robustness	Captures system complexity and uncertainties (e.g., variable patient arrivals, resource availability), allows performance evaluation under realistic scenarios	Faster computation, better planning and scheduling decisions, improved performance metrics (overtime and makespan), superior results versus using simulation alone
Hosseini_Shokouh et al. (2022) [90]	Minimizing patient waiting time and optimize resource (staff, beds) engagement to improve ED efficiency	AI experiments were designed to examine the effect of changes in individuals and equipment on average patient waiting time as well as the engagement rate of the units	Captures complex ED workflows and resource constraints, allowing scenario testing and validation of improvement strategies	Integration enables significant reduction in patient waiting times, improved resource utilization, and more informed, evidence-based resource allocation compared to traditional approaches
Kim et al. (2021) [91]	Developing and optimizing a triage model to minimize mortality rates and improve ICU resource allocation for COVID-19 patients	Provides accurate prognoses to facilitate preemptive treatments, thereby ensuring improvements in patient survival outcomes	Enables evaluation and optimization of triage thresholds under multiple patient influx and resource scenarios, quantifies mortality and resource utilization impacts	Integration enables adaptive, data-driven triage policies that minimize mortality, ensures optimal use of limited ICU resources and supports dynamic response to changing pandemic conditions
Kovalchuk et al. (2018) [92]	Automating and improving simulation of patient flow by identifying and modelling diverse clinical pathways using data-driven methods, improving patient waiting time	Automated, data-driven identification and classification of clinical pathways enable more realistic, granular simulation models and supports personalized care analysis	Allows detailed modelling of complex, multi-department patient flows, resource constraints, and system performance under diverse scenarios	Enables automatic identification of patients’ dynamics on a micro-level, to perform more realistic simulations, and to obtain macro-level characteristics, such as departmental load, queueing parameters, and patients’ experiences
Lazarashouri and Najafi (2024) [93]	Optimizing resource allocation and improving patient flow and service quality in ED	Enables nuanced, robust decision-making that accounts for uncertainty, subjectivity, and multiple conflicting criteria (e.g., balancing urgency, resource limits and service quality)	Allows for risk-free evaluation of operational changes, scenario testing, and quantification of impacts on patient flow and service quality	Integration provides a powerful, adaptable framework for optimizing ED operations: simulation models the system, while fuzzy guides decision-making under uncertainty, leading to significant improvements in patient flow and service quality compared to traditional approach
Lee et al. (2015) [94]	Optimizing allocation of constrained screening resources to maximize early detection of Hepatocellular carcinoma in high-risk patients	Enables adaptive, data-driven allocation of limited screening resources, improving early detection rates and resource efficiency compared to fixed-interval protocols	Allows robust evaluation of screening policies under realistic constraints and patient heterogeneity, quantifying trade-offs and performance metrics	Integration allows for systematic policy optimization and benchmarking, supports learning-based, dynamic resource allocation that outperforms static approach, especially under resource scarcity
Lin et al. (2025) [95]	Assessing the impact of risk-stratified screening scheduling on the daily number of recalls and patient care requirements (e.g., the clinic’s ability to accommodate all scheduled screening mammograms and same-day diagnostics within normal operating hours)	Enables individualized, data-driven risk stratification, allowing for more precise scheduling and recall prediction; supports immediate triage for same-day diagnostics	Quantifies operational impacts of different scheduling templates, evaluates recall variance, patient throughput, waiting times, and required operating hours under realistic workflow constraints	Integration reduces daily recall variance, modestly increases throughput and reduces operating hours, and enables more predictable, efficient workflow for same-day diagnostics compared to traditional scheduling
Mazumdar et al. (2020) [96]	Comparing predictive performance of statistical and AI models for healthcare episode cost prediction in cancer care	Provides greater flexibility and accuracy in cost prediction, especially when data are non-linear, heteroscedastic, or have complex interactions	Enables robust, controlled comparison of model performance across a range of realistic data scenarios	The integration allows for evidence-based selection of predictive models for cost estimation, demonstrating that AI approaches outperform traditional statistical models in most scenarios
Miski et al. (2024) [97]	Automating the generation of simulation models for healthcare systems using natural language processing, and use the resulting simulations to optimize resource allocation (e.g., minimizing costs while meeting patient waiting time constraints)	Automates simulation model generation from natural language, reducing technical barriers and time required for simulation modelling; enables rapid scenario exploration and supports non-expert users in simulation-based decision-making	Enables detailed, risk-free evaluation of resource allocation strategies, patient flow, and operational costs, supporting evidence-based optimization	Integration allows for fast, accurate, and accessible simulation modelling: GPT-4o-generated models produced results comparable to traditional DES, and could optimize resource allocation to minimize costs while maintaining waiting time constraints. This approach accelerates healthcare system modelling and decision support
Nas and Konyuncu (2019) [98]	Optimizing the number of ED beds to minimize patient LoS and improve resource utilization	More accurate prediction of patient arrivals, enabling better-informed resource planning and reducing uncertainty in input parameters	Allows testing of different bed allocation scenarios, quantifies impact on LoS and resource utilization, supports operational and strategic decision-making without real-world risk	Integration leads to more realistic and robust capacity planning by combining accurate, data-driven forecasts with scenario-based evaluation
Ortiz-Barrios et al. (2023) [99]	Predicting the demand for intensive beds and improving several intervention targets such as waiting time for a bed	Accurate prediction of ICU admission likelihood enables early identification of high-risk patients, supports resource planning	Models complex patient flows and resource constraints, allows pre-testing of interventions, quantifies impact on waiting times and resource utilization without real-world risk	Integrating AI predictions with simulation enables scenario analysis for ICU capacity planning, supports rapid, data-driven decision-making, reduces ICU bed waiting times, and improves preparedness for demand surges compared to using AI or simulation alone
Ortiz-Barrios et al. (2024) [100]	Reducing bed waiting times during Seasonal Respiratory Diseases (SRD) peaks by predicting patient risk of respiratory worsening and optimizing resource allocation	Enables accurate, individualized prediction of which ED patients are at high risk for respiratory worsening, supporting proactive resource planning and prioritization	Models complex, dynamic patient flows and resource constraints under SRD peak conditions, allows for evaluation of different bed allocation and resource management strategies to minimize waiting times	Integration allows for data-driven, scenario-based optimization of bed allocation and supports more effective, timely interventions in high-demand periods compared to traditional approaches
Othman et al. (2016) [101]	Minimizing patient waiting time and optimize resource allocation in a paediatric ED considering skill levels and dynamic patient needs	Handles uncertainty, enables real-time negotiation, adapts to dynamic conditions	Simulates paediatric ED operations in real time, supports scenario analysis (e.g., overcrowding, resource shortages), and provides actionable insights for operational improvement	Integration allows for real-time, data-driven management of complex, uncertain paediatric ED environments. This approach improves waiting times, resource use, and care quality compared to traditional scheduling
Sulis et al. (2020) [102]	Optimizing patient admission and scheduling in the ED to minimize LoS and door-to-doctor time (DTDT), maximize throughput, and improve resource utilization	Enables data-driven optimization of admission/scheduling criteria, balances urgency and waiting time, and improves overall system performance (minimizing LoS and DTDT)	Provides detailed, realistic modelling of complex ED dynamics, supports scenario analysis, and visualizes emergent behaviour of agents (patients, staff)	Integration allows for automated, systematic exploration and optimization of decision rules, leading to improved patient flow, reduced waiting times, and more effective resource allocation compared to manual adjustments
Yousefi et al. (2018) [103]	Optimizing resource allocation (doctors, nurses, receptionists) to minimize average LoS and address overcrowding in the ED	Builds a metamodel based on ensemble AI algorithms to investigate input-output relationships of the simulation model. Moreover, after creating the simulation model and making the metamodel, applies an optimization approach to minimize the LoS of patients subject to budget and capacity constraints	Captures complex, stochastic ED dynamics and resource interactions, enabling robust scenario analysis and data generation for metamodel training	Integration allows for rapid, near-optimal resource planning; reduces LoS, enables real-time decision support, and outperforms single-model approaches in both speed and accuracy
Yousefi et al. (2019) [104]	Optimizing allocation of human resources in the ED to minimize DTDT, subject to capacity and budget constraints	Ensemble metamodel provides fast and accurate approximation of simulation outputs, enabling efficient optimization and scenario analysis	Captures complex, dynamic ED operations and human interactions, supports robust scenario testing, and generates data for metamodel training	Integration enables rapid, near-optimal resource allocation, reduces DTDT, and identifies bottlenecks with less computational time compared to direct simulation-optimization

## Data Availability

No new data was created.

## Abbreviations

The following abbreviations are used in this manuscript:

ABS	Agent-Based Simulation
AI	Artificial Intelligence
ANFIS	Adaptive Neuro-Fuzzy Inference System
BN	Bayesian Network
CNN	Convolutional Neural Network
CXR	Chest X-Ray
DES	Discrete Event Simulation
DR	Decision Rules
DT	Decision Tree
DTDT	Door-to-Doctor Time
ED	Emergency Department
FIFO	First In, First Out
FNN	Feedforward Neural Network
GA	Genetic Algorithm
GB	Gradient Boosting
ICU	Intensive Care Unit
KNN	K-Nearest Neighbours
LLM	Large Language Model
LoS	Length of Stay
LR	Logistic Regression
ML	Machine Learning
MMAT	Mixed Methods Appraisal Tool
MLP	Multilayer Perceptron
NB	Naïve Bayes
NN	Neural Network
RF	Random Forest
RNN	Recurrent Neural Network
RTAT	Report Turnaround Time
SG	Stochastic Gradient regression
SRD	Seasonal Respiratory Diseases
SVM	Support Vector Machine

## References

[1] Van Zyl-Cillié M., Demirtas D., Hans E. (2023). Wait! What does that mean?: Eliminating ambiguity of delays in healthcare from an OR/MS perspective. Health Syst..

[2] Emilio C., de Pablos L., Rodriguez M.V., Hall R. (2013). Waiting Lists for Surgery. Patient Flow: Reducing Delay in Healthcare Delivery.

[3] Pandey M.K., Gangeshwer D.K. (2023). Application of Queuing Theory to Analysis of Waiting Time in the Hospital. Int. J. Bioautom..

[4] Long J., Tan E.D., Xu Z. (2024). Optimization Study of Medical Waiting Time from the Perspective of Behavioral Economics Take Canada as an Example. Adv. Econ. Manag. Political Sci..

[5] Pringgayuda F., Hashim F., Alsaidi N.A.Y. (2022). Waiting Time of Patients in Outpatient Hospital Before and After Pandemic Covid 19: A Literature Review. J. Health Sci. Prev..

[6] Shah S.A., Jeffrey K., Robertson C., Sheikh A. (2025). Impact of COVID-19 pandemic on elective care backlog trends, recovery efforts, and capacity needs to address backlogs in Scotland (2013–2023): A descriptive analysis and modelling study. Lancet Reg. Health Eur..

[7] Uimonen M., Kuitunen I., Paloneva J., Launonen A.P., Ponkilainen V., Mattila V.M. (2021). The impact of the COVID-19 pandemic on waiting times for elective surgery patients: A multicenter study. PLoS ONE.

[8] van den Broek A., de Vroege L. (2024). Don’t Postpone! Mental Health of Healthcare Professionals Needs Attention!. Explor. Res. Hypothesis Med..

[9] Ward P.R., Rokkas P., Cenko C., Pulvirenti M., Dean N., Carney A.S., Meyer S. (2017). “Waiting for” and “waiting in” public and private hospitals: A qualitative study of patient trust in South Australia. BMC Health Serv. Res..

[10] Naiker U., FitzGerald G., Dulhunty J.M., Rosemann M. (2018). Time to wait: A systematic review of strategies that affect out-patient waiting times. Aust. Health Rev..

[11] Hosny A., Parmar C., Quackenbush J., Schwartz L.H., Aerts H.J.W.L. (2018). Artificial intelligence in radiology. Nat. Rev. Cancer.

[12] Piorkowski A., Obuchowicz R., Najjar R. (2023). Redefining Radiology: A Review of Artificial Intelligence Integration in Medical Imaging. Diagnostics.

[13] Sorantin E., Grasser M.G., Hemmelmayr A., Tschauner S., Hrzic F., Weiss V., Lacekova J., Holzinger A. (2021). The augmented radiologist: Artificial intelligence in the practice of radiology. Pediatr. Radiol..

[14] Niazi M.K.K., Parwani A.V., Gurcan M.N. (2019). Digital pathology and artificial intelligence. Lancet Oncol..

[15] Song A.H., Jaume G., Williamson D.F.K., Lu M.Y., Vaidya A., Miller T.R., Mahmood F. (2023). Artificial intelligence for digital and computational pathology. Nat. Rev. Bioeng..

[16] Baxi V., Edwards R., Montalto M., Saha S. (2022). Digital pathology and artificial intelligence in translational medicine and clinical practice. Mod. Pathol..

[17] Johnson K.W., Torres Soto J., Glicksberg B.S., Shameer K., Miotto R., Ali M., Ashley E., Dudley J.T. (2018). Artificial Intelligence in Cardiology. J. Am. Coll. Cardiol..

[18] Karatzia L., Aung N., Aksentijevic D. (2022). Artificial intelligence in cardiology: Hope for the future and power for the present. Front. Cardiovasc. Med..

[19] Ledziński Ł., Grześk G. (2023). Artificial Intelligence Technologies in Cardiology. J. Cardiovasc. Dev. Dis..

[20] Gonem S., Janssens W., Das N., Topalovic M. (2020). Applications of artificial intelligence and machine learning in respiratory medicine. Thorax.

[21] Casal-Guisande M., Mosteiro-Añón M., Torres-Duran M., Comesaña-Campos A., Fernández-Villar A. (2025). Application of artificial intelligence for the detection of obstructive sleep apnea based on clinical and demographic data: A systematic review. Expert Rev. Respir. Med..

[22] Casal-Guisande M., Villar-Aguilar L., Fernández-Villar A., García-Rodríguez E., Casal A., Torres-Durán M. (2025). Applications of Artificial Intelligence in Alpha-1 Antitrypsin Deficiency: A Systematic Review from a Respiratory Medicine Perspective. Medicina.

[23] Casal-Guisande M., Represas-Represas C., Golpe R., Fernández-García A., González-Montaos A., Comesaña-Campos A., Ruano-Raviña A., Fernández-Villar A. (2024). Clinical and Social Characterization of Patients Hospitalized for COPD Exacerbation Using Machine Learning Tools. Arch. Bronconeumol..

[24] Villar-Aguilar L., Casal-Guisande M., Fernández-Villar A., García-Rodríguez E., Priegue-Carrera A., Miravitlles M., Torres-Durán M. (2025). Characterisation of patients with Alpha-1 antitrypsin deficiency using unsupervised machine learning tools. Respir. Med..

[25] López-Canay J., Casal-Guisande M., Pinheira A., Golpe R., Comesaña-Campos A., Fernández-García A., Represas-Represas C., Fernández-Villar A. (2025). Predicting COPD Readmission: An Intelligent Clinical Decision Support System. Diagnostics.

[26] Casal-Guisande M., Represas-Represas C., Golpe R., Comesaña-Campos A., Fernández-García A., Torres-Durán M., Fernández-Villar A. (2025). Improving End-of-Life Care for COPD Patients: Design and Development of an Intelligent Clinical Decision Support System to Predict One-Year Mortality After Acute Exacerbations. Int. J. Intell. Syst..

[27] Casal-Guisande M., Comesaña-Campos A., Núñez-Fernández M., Torres-Durán M., Fernández-Villar A. (2024). Proposal and Definition of an Intelligent Clinical Decision Support System Applied to the Prediction of Dyspnea after 12 Months of an Acute Episode of COVID-19. Biomedicines.

[28] Esteva A., Kuprel B., Novoa R.A., Ko J., Swetter S.M., Blau H.M., Thrun S. (2017). Dermatologist-level classification of skin cancer with deep neural networks. Nature.

[29] Rajpurkar P., Irvin J., Zhu K., Yang B., Mehta H., Duan T., Ding D., Bagul A., Langlotz C., Shpanskaya K. (2017). CheXNet: Radiologist-Level Pneumonia Detection on Chest X-Rays with Deep Learning. arXiv.

[30] Topol E.J. (2019). High-performance medicine: The convergence of human and artificial intelligence. Nat. Med..

[31] Miotto R., Wang F., Wang S., Jiang X., Dudley J.T. (2018). Deep learning for healthcare: Review, opportunities and challenges. Brief. Bioinform..

[32] Erickson B.J., Korfiatis P., Akkus Z., Kline T.L. (2017). Machine Learning for Medical Imaging. RadioGraphics.

[33] Al-Mousa A., Al-Zubaidi H., Al-Dweik M. (2024). A machine learning-based approach for wait-time estimation in healthcare facilities with multi-stage queues. IET Smart Cities.

[34] Cinaroglu S. (2016). Complexity in healthcare management: Why does Drucker describe healthcare organizations as a double-headed monster?. Int. J. Healthc. Manag..

[35] Bosilj Vukšić V., Bach M.P., Tomičić-Pupek K. (2017). Utilization of Discrete Event Simulation in Business Processes Management Projects: A Literature Review. J. Inf. Organ. Sci..

[36] Crespo-Pereira D., Pérez-Rodríguez A., Carballo-Alonso A., Costas-Freire D., Rodal-Salgueiro A., Iglesias-Fernández S., del-Río-Vilas D., Carrillo-Lasheras A. (2021). Maven: A first step towards a digital twin for synchronous logistics in the automotive industry. Proceedings of the 20th International Conference on Modeling and Applied Simulation, MAS 2021.

[37] Ghosh A., Dey D. (2024). Digital Twin for EEG seizure prediction using time reassigned Multisynchrosqueezing transform-based CNN-BiLSTM-Attention mechanism model. Biomed. Phys. Eng. Express.

[38] Förster K.M., Roth C.J., Hilgendorff A., Ertl-Wagner B., Flemmer A.W., Wall W.A. (2021). In silico numerical simulation of ventilator settings during high-frequency ventilation in preterm infants. Pediatr. Pulmonol..

[39] Vanbrabant L., Braekers K., Ramaekers K., Van Nieuwenhuyse I. (2019). Simulation of emergency department operations: A comprehensive review of KPIs and operational improvements. Comput. Ind. Eng..

[40] Doneda M., Yalçındağ S., Marques I., Lanzarone E. (2021). A discrete-event simulation model for analysing and improving operations in a blood donation centre. Vox Sang..

[41] Ponsiglione A.M., Romano M., Amato F. (2021). A Finite-State Machine Approach to Study Patients Dropout from Medical Examinations. Proceedings of the 6th International Forum on Research and Technology for Society and Industry, RTSI 2021—Proceedings.

[42] Yip K., Leung L., Yeung D. (2018). Levelling bed occupancy: Reconfiguring surgery schedules via simulation. Int. J. Health Care Qual. Assur..

[43] Pellegrino M., Lombardo G., Poggi A. (2024). Towards Digital Twins in Healthcare: Optimizing Operating Room and Recovery Room Dynamics. Procedia Comput. Sci..

[44] Isiaka S.O., Babatunde R.S., Isiaka R.M. (2024). Exploring Artificial Intelligence (AI) Technologies in Predictive Medicine: A Systematic Review. Kasu J. Comput. Sci..

[45] Alvarado M., Lawley M., Li Y. Healthcare simulation tutorial: Methods, challenges, and opportunities. Proceedings of the 2016 Winter Simulation Conference (WSC).

[46] von Rueden L., Mayer S., Sifa R., Bauckhage C., Garcke J., Berthold M.R., Feelders A., Krempl G. (2020). Combining Machine Learning and Simulation to a Hybrid Modelling Approach: Current and Future Directions. Advances in Intelligent Data Analysis XVIII.

[47] Shi Y., Mahdian S., Blanchet J., Glynn P., Shin A.Y., Scheinker D. (2023). Surgical Scheduling via Optimization and Machine Learning with Long-Tailed Data: Health Care Management Science. Health Care Manag. Sci..

[48] Alharbi L.A. (2023). Artificial Rabbits Optimizer With Machine Learning Based Emergency Department Monitoring and Medical Data Classification at KSA Hospitals. IEEE Access.

[49] Juliet S.D., Banumathi J. (2025). Prescriptive analytics decision-making system for cardiovascular disease prediction in long COVID patients using advanced reinforcement learning algorithms. J. X-Ray Sci. Technol. Clin. Appl. Diagn. Ther..

[50] Othman S., Ben Hammadi S., Quilliot A., Martinot A., Renard J.M. (2015). Health Care Decision Support System for the Pediatric Emeregency Department Management. MEDINFO 2015: eHealth-Enabled Health.

[51] Safdari R., Malak S.S., Mohammadzadeh J., Shahraki D., Multi A. (2017). Agent Based Approach for Prehospital Emergency Management. Bull. Emerg. Trauma.

[52] Huang S.-W., Weng S.-J., Chiou S.-Y., Nguyen T.-D., Chen C.-H., Liu S.-C., Tsai Y.-T. (2024). A Study on Decision-Making for Improving Service Efficiency in Hospitals. Healthcare.

[53] Ponsiglione A.M., Zaffino P., Ricciardi C., Di Laura D., Spadea M.F., De Tommasi G., Improta G., Romano M., Amato F. (2024). Combining simulation models and machine learning in healthcare management: Strategies and applications. Prog. Biomed. Eng..

[54] Entezari B., Koucheki R., Abbas A., Toor J., Wolfstadt J.I., Ravi B., Whyne C., Lex J.R. (2023). Improving Resource Utilization for Arthroplasty Care by Leveraging Machine Learning and Optimization: A Systematic Review. Arthroplast. Today.

[55] Page M.J., McKenzie J.E., Bossuyt P.M., Boutron I., Hoffmann T.C., Mulrow C.D., Shamseer L., Tetzlaff J.M., Akl E.A., Brennan W.E. (2021). The PRISMA 2020 statement: An updated guideline for reporting systematic reviews. BMJ.

[56] Page M.J., Moher D., Bossuyt P.M., Boutron I., Hoffmann T.C., Mulrow C.D., Shamseer L., Tetzlaff J.M., Akl E.A., Brennan W.E. (2021). PRISMA 2020 explanation and elaboration: Updated guidance and exemplars for reporting systematic reviews. BMJ.

[57] Hong Q.N., Pluye P., Fàbregues S., Bartlett G., Boardman F., Cargo M., Dagenais P., Gagnon M.-P., Griffiths F., Nicolau B. (2019). Improving the content validity of the mixed methods appraisal tool: A modified e-Delphi study. J. Clin. Epidemiol..

[58] Hong Q.N., Pluye P., Fabregues S., Bartlett G., Boardman F., Cargo M., Dagenais P., Gagnon M.-P., Griffiths F., Nicolau B. (2018). Mixed Methods Appraisal Tool (MMAT) Version 2018 User Guide.

[59] Guido R., Conforti D. (2017). A hybrid genetic approach for solving an integrated multi-objective operating room planning and scheduling problem. Comput. Oper. Res..

[60] Renc P., Jia Y., Samir A.E., Was J., Li Q., Bates D.W., Sitek A. (2024). Zero shot health trajectory prediction using transformer. NPJ Digit. Med..

[61] Baesler F., Gatica J., Correa R. (2015). Simulation optimisation for operating room scheduling. Int. J. Simul. Model..

[62] M’Hallah R., Al-Roomi A.H. (2014). The planning and scheduling of operating rooms: A simulation approach. Comput. Ind. Eng..

[63] Chemkomnerd N., Pannakkong W., Tanantong T., Huynh V.-N., Karnjana J. (2024). A Simulation-Based Multi-Objective Optimization Framework to Enhance Patient Satisfaction: A Case Study of Ophthalmology Department Management. IEEE Access.

[64] Bruballa E., Wong A., Rexachs D., Luque E. (2020). An intelligent scheduling of non-critical patients admission for emergency department. IEEE Access.

[65] Gehlot V., King D., Schaffer J., Sloane E.B., Wickramasinghe N. (2022). Healthcare Optimization and Augmented Intelligence by Coupling Simulation & Modeling: An Ideal AI/ML Partnership for a Better Clinical Informatics. AMIA Annu. Symp. Proc..

[66] Lin Y., Hoyt A.C., Manuel V.G., Inkelas M., Maehara C.K., Ayvaci M.U.S., Ahsen M.E., Hsu W. (2025). Integrating AI into Clinical Workflows: A Simulation Study on Implementing AI-aided Same-day Diagnostic Testing Following an Abnormal Screening Mammogram. AMIA Annu. Symp. Proc..

[67] Thompson Y.L.E., Levine G.M., Chen W., Sahiner B., Li Q., Petrick N., Delfino J.G., Lago M.A., Cao Q., Samuelson F.W. (2024). Applying queueing theory to evaluate wait-time-savings of triage algorithms. Queueing Syst..

[68] Lin Y., Hoyt A.C., Manuel V.G., Inkelas M., Hsu W. (2024). Using Discrete Event Simulation to Design and Assess an AI-aided Workflow for Same-day Diagnostic Testing of Women Undergoing Breast Screening. AMIA Jt. Summits Transl. Sci. Proc..

[69] Wornow M., Gyang Ross E., Callahan A., Shah N.H. (2023). APLUS: A Python library for usefulness simulations of machine learning models in healthcare. J. Biomed. Inform..

[70] Srinivas S., Salah H. (2021). Consultation length and no-show prediction for improving appointment scheduling efficiency at a cardiology clinic: A data analytics approach. Int. J. Med. Inform..

[71] Zhu D.D., Sun J.Q., Zhao Y. (2021). A Hybrid GA-SA for the Urgent Patients Disturbed Physical Examination Rescheduling Problem Considering Setup Time. IEEE Access.

[72] Rogers P., Boussina A.E., Shashikumar S.P., Wardi G., Longhurst C.A., Nemati S. (2023). Optimizing the Implementation of Clinical Predictive Models to Minimize National Costs: Sepsis Case Study. J. Med. Internet Res..

[73] Hunter-Zinck H.S., Peck J.S., Strout T.D., Gaehde S.A. (2019). Predicting emergency department orders with multilabel machine learning techniques and simulating effects on length of stay. J. Am. Med. Inform. Assoc..

[74] Williams A., Mekhail A.M., Williams J., McCord J., Buchan V. (2019). Effective resource management using machine learning in medicine: An applied example. BMJ Simul. Technol. Enhanc. Learn..

[75] Devarajan J.P., Manimuthu A., Sreedharan V.R. (2023). Healthcare Operations and Black Swan Event for COVID-19 Pandemic: A Predictive Analytics. IEEE Trans. Eng. Manag..

[76] Roy S., Dutta R., Ghosh P. (2021). Towards Dynamic lockdown strategies controlling pandemic spread under healthcare resource budget. Appl. Netw. Sci..

[77] Yao Y., Zhou H., Cao Z., Zeng D.D., Zhang Q. (2023). Optimal adaptive nonpharmaceutical interventions to mitigate the outbreak of respiratory infections following the COVID-19 pandemic: A deep reinforcement learning study in Hong Kong, China. J. Am. Med. Inform. Assoc..

[78] Sulis E., Terna P. (2021). An Agent-based Decision Support for a Vaccination Campaign. J. Med. Syst..

[79] Diaz-Milanes D., Almeda N., Gutierrez-Colosia M.R., Garcia-Alonso C.R., Sadeniemi M., Salvador-Carulla L. (2023). Impact of the workforce allocation on the technical performance of mental health services: The collective case of Helsinki-Uusimaa (Finland). Health Res. Policy Syst..

[80] Diaz-Milanes D., Almeda N., Rodero-Cosano M.L., Salinas-Perez J.A., Garcia-Alonso C.R. (2024). Assessment of care provision integration in a community-based mental health system: Balanced care model implementation in Andalusia (Spain). BMC Public Health.

[81] García-Alonso C.R., Almeda N., Salinas-Pérez J.A., Gutiérrez-Colosía M.R., Iruin-Sanz Á., Salvador-Carulla L. (2022). Use of a decision support system for benchmarking analysis and organizational improvement of regional mental health care: Efficiency, stability and entropy assessment of the mental health ecosystem of Gipuzkoa (Basque Country, Spain). PLoS ONE.

[82] García-Alonso C.R., Almeda N., Salinas-Pérez J.A., Gutiérrez-Colosía M.R., Uriarte-Uriarte J.J., Salvador-Carulla L. (2019). A decision support system for assessing management interventions in a mental health ecosystem: The case of Bizkaia (Basque Country, Spain). PLoS ONE.

[83] Abuhay T.M., Robinson S., Mamuye A., Kovalchuk S.V. (2023). Machine learning integrated patient flow simulation: Why and how?. J. Simul..

[84] Allen M., James C., Frost J., Liabo K., Pearn K., Monks T., Zhelev Z., Logan S., Everson R., James M. (2022). Using simulation and machine learning to maximise the benefit of intravenous thrombolysis in acute stroke in England and Wales: The SAMueL modelling and qualitative study. Health Soc. Care Deliv. Res..

[85] Atalan A., Şahin H., Atalan Y.A. (2022). Integration of Machine Learning Algorithms and Discrete-Event Simulation for the Cost of Healthcare Resources. Healthcare.

[86] Baltruschat I., Steinmeister L., Nickisch H., Saalbach A., Grass M., Adam G., Knopp T., Ittrich H. (2021). Smart chest X-ray worklist prioritization using artificial intelligence: A clinical workflow simulation. Eur. Radiol..

[87] De Deken V.J., Cools W., Van Deynse H., Putman K., Barbé K. (2025). A Markov Chain methodology for care pathway mapping using health insurance data, a study case on pediatric TBI. Comput. Methods Programs Biomed..

[88] Gartner D., Padman R. (2020). Machine learning for healthcare behavioural OR: Addressing waiting time perceptions in emergency care. J. Oper. Res. Soc..

[89] Hadid M., Elomri A., Padmanabhan R., Kerbache L., Jouini O., El Omri A., Nounou A., Hamad A. (2022). Clustering and Stochastic Simulation Optimization for Outpatient Chemotherapy Appointment Planning and Scheduling. Int. J. Environ. Res. Public. Health.

[90] Hosseini-Shokouh S.M., Mohammadi K., Yaghoubi M. (2022). Optimization of Service Process in Emergency Department Using Discrete Event Simulation and Machine Learning Algorithm. Arch. Acad. Emerg. Med..

[91] Kim J., Lim H., Ahn J.H., Lee K.H., Lee K.S., Koo K.C. (2021). Optimal triage for covid-19 patients under limited health care resources with a parsimonious machine learning prediction model and threshold optimization using discrete-event simulation: Development study. JMIR Med. Inform..

[92] Kovalchuk S.V., Funkner A.A., Metsker O.G., Yakovlev A.N. (2018). Simulation of patient flow in multiple healthcare units using process and data mining techniques for model identification. J. Biomed. Inform..

[93] Lazarashouri H., Najafi S.E. (2024). Enhancing Emergency Department Efficiency Through Simulation and Fuzzy Multi-Criteria Decision-Making Integration. J. Oper. Strateg. Anal..

[94] Lee E., Lavieri M.S., Volk M.L., Xu Y. (2015). Applying reinforcement learning techniques to detect hepatocellular carcinoma under limited screening capacity. Health Care Manag. Sci..

[95] Lin Y., Hoyt A.C., Manuel V.G., Inkelas M., Ayvaci M.U.S., Ahsen M.E., Hsu W. (2025). Risk-Stratified Screening: A Simulation Study of Scheduling Templates on Daily Mammography Recalls. J. Am. Coll. Radiol..

[96] Mazumdar M., Lin J.Y.J., Zhang W., Li L., Liu M., Dharmarajan K., Sanderson M., Isola L., Hu L. (2020). Comparison of statistical and machine learning models for healthcare cost data: A simulation study motivated by Oncology Care Model (OCM) data. BMC Health Serv. Res..

[97] Miski A., Correspondence A., Sharif A.T., Bukhari A.G., Ismail M. (2024). Modeling Discrete-Event Simulations Using Natural Language Processing: A Healthcare Application Modeling Discrete-Event Simulations Using Natural Language Processing: A Healthcare Application Modeling Discrete-Event Simulations Using Natural Language Processing: A Healthcare Application. J. King Abdulaziz Univ. Med. Sci..

[98] Nas S., Koyuncu M. (2019). Emergency Department Capacity Planning: A Recurrent Neural Network and Simulation Approach. Comput. Math. Methods Med..

[99] Ortiz-Barrios M., Arias-Fonseca S., Ishizaka A., Barbati M., Avendaño-Collante B., Navarro-Jiménez E. (2023). Artificial intelligence and discrete-event simulation for capacity management of intensive care units during the Covid-19 pandemic: A case study. J. Bus. Res..

[100] Ortiz-Barrios M., Ishizaka A., Barbati M., Arias-Fonseca S., Khan J., Gul M., Yücesan M., Alfaro-Saíz J.-J., Pérez-Aguilar A. (2024). Integrating discrete-event simulation and artificial intelligence for shortening bed waiting times in hospitalization departments during respiratory disease seasons. Comput. Ind. Eng..

[101] Ben Othman S., Zgaya H., Hammadi S., Quilliot A., Martinot A., Renard J.M. (2016). Agents endowed with uncertainty management behaviors to solve a multiskill healthcare task scheduling. J. Biomed. Inform..

[102] Sulis E., Terna P., Di Leva A., Boella G., Boccuzzi A. (2020). Agent-oriented Decision Support System for Business Processes Management with Genetic Algorithm Optimization: An Application in Healthcare. J. Med. Syst..

[103] Yousefi M., Yousefi M., Ferreira R.P.M., Kim J.H., Fogliatto F.S. (2018). Chaotic genetic algorithm and Adaboost ensemble metamodeling approach for optimum resource planning in emergency departments. Artif. Intell. Med..

[104] Yousefi M., Yousefi M. (2020). Human resource allocation in an emergency department: A metamodel-based simulation optimization. Kybernetes.

